# Untimely Exercise

**Published:** 1858-11

**Authors:** 


### UNTIMELY EXERCISE.

A brisk walk in a cool, bracing atmosphere, is a luxury, provided it be taken under proper circumstances. SIDNEY SMITH made a great mistake, when he said that a public speaker would never break down, if he would walk a dozen miles before speaking. He might not break down in one sense of the word, for there would be nothing to break; he would have no strength, and there would be no elevation to tumble from, because his speech would be as flat as cold soup. The less a man exercises before a morning's sermon, or speech, the better. The vital energy should not be expended on the muscles, but on the brain. To speak with freshness, and with a vigor which shall carry all before it, a man should neither sing, talk, nor walk before speaking.

